# Anemia and leukopenia following intravenous colloidal silver infusions—Clinical and hematological features, unique peripheral blood film appearance and effective therapy with supplemental oral copper and apheresis

**DOI:** 10.1002/ccr3.2316

**Published:** 2019-08-09

**Authors:** Ethan A. Natelson, Kelty R. Baker, David W. Pyatt

**Affiliations:** ^1^ Department of Clinical Medicine Weill Cornell Medical College New York NY USA; ^2^ Department of Academic Medicine Houston Methodist Hospital, Methodist Hospital Research Institute Houston TX USA; ^3^ Houston Methodist Hospital Houston TX USA; ^4^ School of Public Health University of Colorado Boulder CO USA; ^5^ School of Pharmacy University of Colorado Boulder CO USA; ^6^ Summit Technology, LLP West Hartford CT USA

**Keywords:** apheresis, colloidal silver, copper, ineffective myelopoiesis

## Abstract

Alternative medical therapy with multiple intravenous colloidal silver infusions may cause severe illness, including profound copper deficiency‐induced anemia and hepatic toxicity. No chelating agent for silver poisoning exists and effective therapy requires apheresis in combination with continuous administration of oral copper.

## 1 INTRODUCTION

Three patients received purported therapeutic medical treatment at a Wellness Center, including multiple intravenous infusions containing colloidal silver. We describe clinical consequences, laboratory features, and results of corrective therapy for ensuing and persistent anemia with the combination of oral copper sulfate administration and therapeutic apheresis in one of these patients.

The potential toxicities of colloidal silver‐containing preparations administered topically, or taken orally, are well‐known, but little published information is available concerning the adverse health effects of intravenously administered colloidal silver solutions. We report here, a group of three patients receiving alternative and purported therapeutic medical treatment at a Wellness Center, which included multiple intravenous colloidal silver infusions. We describe the clinical consequences and corrective therapy for the ensuing and persistent anemia with the combination of oral copper sulfate administration and therapeutic apheresis, in one of these patients. This outcome contrasts with the continuing anemia and disability in the other two patients, who declined apheresis.

Pharmaceutical preparations and bandages containing silver salts or colloidal silver have long been used topically in patients with burns and other wounds as a protection barrier against infection.^1^ However, silver‐containing compounds have never been proven as effective systemic therapy against any specific pathogens either by oral or parenteral administration.^2^, ^3^ A Federal Drug Administration (FDA) Advisory Expert Panel found no efficacy in humans for its systemic administration and silver as medication was long ago removed from its approved use in over the counter product publications.^2^ The excretion of absorbed oral silver is very poor—some may be lost through shedding intestinal cellular material and in the bile but only very small amounts appear in the urine.^2^, ^3^ However, there appears to be no significant excretory pathway for colloidal silver administered intravenously and a severe and unusual form of persistent anemia and some degree of neutropenia is a consequence.

A dreaded complication of silver poisoning is argyria, a gray‐blue discoloration of the skin and present even in the sclera, and that is permanent.^2^, ^4^, ^5^ Other potential systemic complications associated with silver toxicity include neurological, hepatic, and renal aberrations that may cause disability or death.^2^, ^6^, ^7^ No chelating agents have yet proven useful in remitting silver toxicity, and this includes more recently introduced products such as dimercaptosuccinic acid (Succimer), useful in lead poisoning.^8^ The mechanism resulting in anemia consequent to intravenous silver infusion is, in large part, due to accelerated, persistent, and profound copper and perhaps zinc excretory losses promoted by the elevated serum silver levels.^9^, ^10^ Leukopenia and neutropenia may also be present but seem less of a clinically important issue than the anemia.

## 2 CASE PRESENTATION

### 2.1 Case 1

A 30‐year‐old woman was referred to our hematology section because of a severe anemia. Several months earlier, she had sought medical attention at a Wellness Center for symptoms of fatigue and depression. She denied suffering weight loss, tick bites, skin rash, or fever. Her evaluation included numerous laboratory diagnostic studies including an IgG panel consisting of 10 separate markers targeting Lyme disease antigens along with a similar panel for IgM markers. All of these results were normal as was a Western blot assay for Lyme disease. A routine blood count showed a hemoglobin concentration of 14.4 g/dL, along with a total leukocyte count of 7.5 × 10^9^/L and platelet count of 257 × 10^9^/L. A differential cell count included 66.7% neutrophils, 21.8% lymphocytes, and 9.8% monocytes. Despite this extensive laboratory evaluation, she was informed that she was suffering from Lyme disease and required specialized therapy. This treatment consisted of 48 separate infusions, each containing hydrogen peroxide, ozone, and colloidal silver administered through a peripherally inserted central catheter (PIC line), over a 3‐month interval. About midway during this treatment period, another blood count indicated her hemoglobin concentration had fallen to 10.9 g/dL with a total leukocyte count of 3.1 × 10^9^/L with 32% neutrophils, 39% lymphocytes, and 25% monocytes and a normal platelet count.

Two months after completing this treatment plan, she noticed progressive fatigue and ultimately sought care at an emergency center. Her hemoglobin concentration was 6 g/dL with a mean corpuscular volume (MCV) of 97 fL, a reduced white blood cell count of 1.7 × 10^9^/L with neutropenia, and a normal platelet count. She received three units of packed red blood cells in treatment of the anemia and a single injection of Neupogen, 300 mcg, to improve her neutropenia. She underwent a bone marrow study several days later. Her blood count then showed a hemoglobin concentration of 8.7 g/dL, with a total leukocyte count of 1.5 × 10^9^/L and platelet count of 318 × 10^9^/L.

We obtained and reviewed the bone marrow slide material and agreed with the hematopathology report, which described a 60% cellular marrow with 1% blasts, 31% myelocytes/metamyelocytes, 15% bands and segmented neutrophils, 4% eosinophils, 19% erythroid cells, 9% monocytes, 13% lymphocytes, including hematogones, and 19% erythroid precursors with normoblastic maturation and no significant dysplasia. While hematogones were increased, there was no increase in myeloblasts. There was mild megakaryocytic hyperplasia. The myeloid to erythroid (M:E) cell ratio was 2.7. A flow cytometry cell differential showed 30.9% lymphocytes, 14% monocytes, and a total of 42.8% granulocytes and no increase in phenotypic blast forms. Iron stores were increased but ringed sideroblasts were not identified. There was no evident erythrophagocytosis by histiocytes. Cytogenetic studies gave normal results. No specific diagnosis was suggested, and a clinical hematology consultation was recommended.

**Figure 1:**
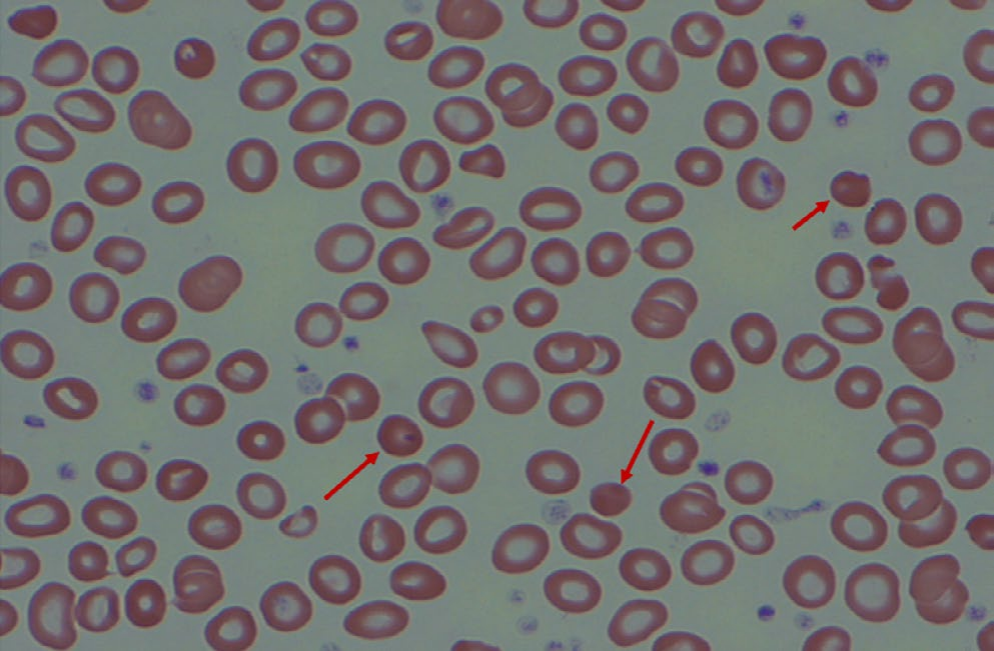
Variation in erythrocyte shape, size and coloration (poikilocytosis and anisochromia) with spherocytes (arrows) and leukopenia

**Figure 2:**
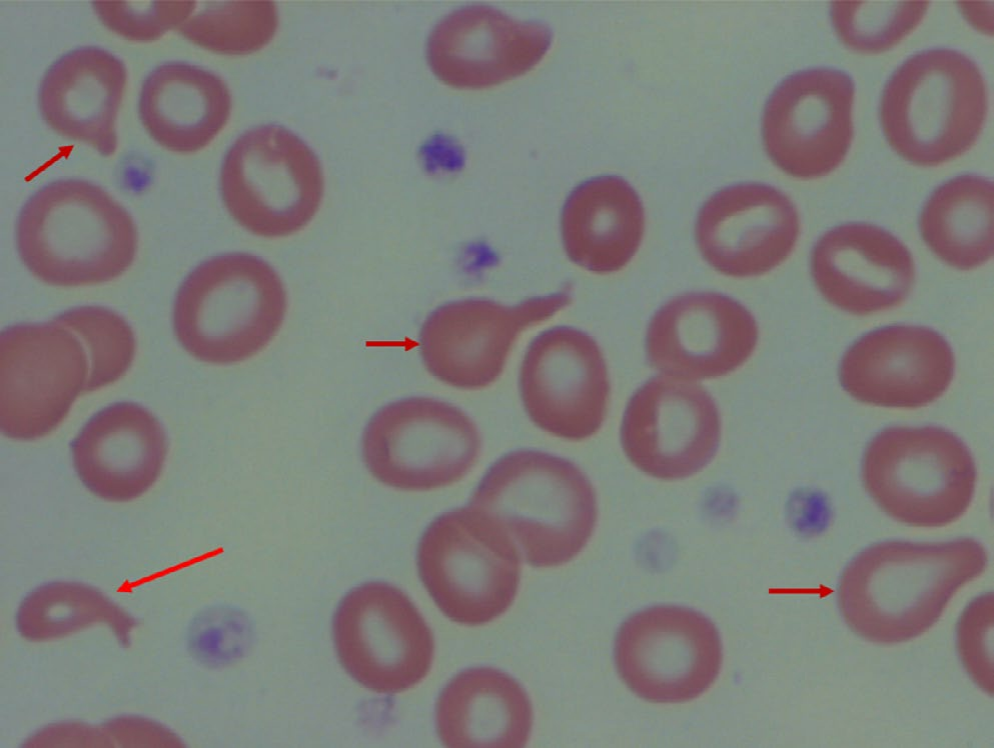
Tear drop erythrocytes or dacrocytes (arrows)

**Figure 3:**
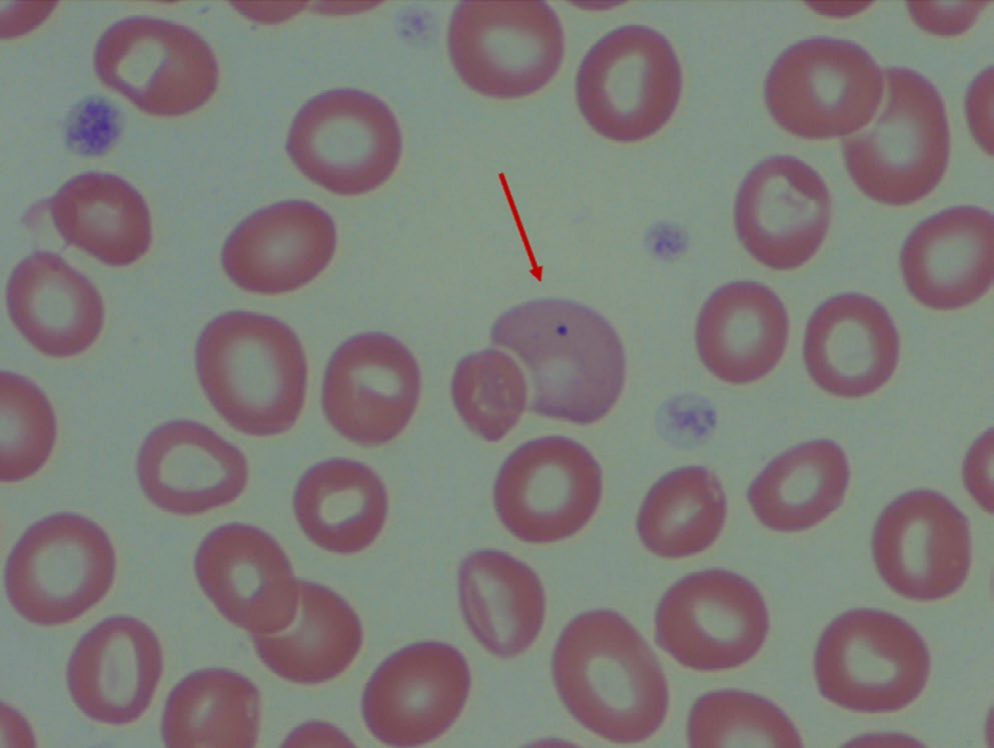
Polychromatophilic erythrocyte containing a Howell Jolly body (arrow)

Her initial consultative blood count, obtained several weeks after the blood transfusion and bone marrow study, showed a hemoglobin concentration of 9.0 g/dL with a total leukocyte count of 2.3 × 10^9^/L with 50% neutrophils. The MCV was 97 fL, and the platelet count was normal. Her peripheral blood film was extremely abnormal containing large numbers of tear drop erythrocytes (dacrocytes) of varying size and shape along with marked poikilocytosis and anisochromia (Figures 1 and 2). A few large, polychromatic erythrocytes contained Howell‐Jolly bodies (Figure 3). There was also a very small but definitive population of spherocytes, but no microangiopathic changes. Basophilic stippling was not noted, and circulating normoblasts were not present—mature neutrophil and platelet morphology were unremarkable. The serum ferritin value, normal on her pretreatment screening, was elevated at 500 ng/dL. The blood lead level was <2.0 mcg/dL (normal range, up to 4.9 mcg/dL). The serum copper concentration was <5 mcg/dL.

We ultimately obtained her prior medical records and then first learned that she had received the specific colloidal silver preparation Argentyn 23, marketed for oral usage, in her infusions. This particular product contains 236 mg Ag/L. She received variable volume amounts of the Argentyn 23 solution with each infusion, from 20 mL to 180 mL, without explanation. Accordingly, her total dose of parenteral silver amounted to 883 mg.

Initially, she received treatment for her continuing anemia and profound copper deficiency with 12 mg of copper sulfate daily in oral divided doses with meals. Her hemoglobin concentration improved slightly, and the leukopenia resolved over a 2‐month period but serum copper measurements remained below accurate quantitation levels. Her serum silver level also remained unchanged from its initial value, at 350 mcg/L. After a few additional months of observation, and realizing that there was to be no progressive fall in the markedly elevated silver level, or correction of the greatly reduced serum copper level, despite oral supplements, we began apheresis therapy. This process is accomplished at our blood center with automated equipment designed to exchange 1‐1.5 of the patient's total plasma volume with 5% albumin in normal saline, containing calcium gluconate and potassium chloride. After the first plasma exchange, the serum copper became accurately measurable at 21 mcg/L (normal range 80‐155 mcg/L), after the second exchange, it was 40 mcg/L, after the third exchange 50 mcg/L,and after the fourth exchange, it reached 79 mcg/L.

Her hemoglobin concentration slowly rose to 13.4 g/dL, and her peripheral blood film became entirely normal in appearance. We had allowed a few weeks between each apheresis. Her serum silver level fell to 240 mcg/L after the first apheresis, to 200 mcg/L after the second, to 160 mcg/L after the third exchange, and to 130 mcg/L after the fourth exchange. At that point, we reduced her oral copper intake to 4 mg daily and decided to simply observe subsequent laboratory values and her clinical status. Her initially elevated serum ferritin value had fallen to 165 ng/mL. A psychiatrist successfully treated her depression with an oral medication, at low dosage, and she remains asymptomatic.

While evaluating and discussing this patient with other hematologists, one of the authors (KRB) reported that she had recently been caring for two women, one who was quite ill and admitted to a nearby medical center hospital. Both had been treated in the same fashion as my patient, and at the same Wellness Center and for the same alleged diagnosis of Lyme disease. These patients will be described as Case 2 and Case 3.

### 2.2 Case 2

A 74‐year‐old woman with no past history of anemia sought medical attention in another city and received multiple antibiotics for 2 months as treatment for alleged Lyme disease. Returning to her home, she presented to the Wellness Center discussed in this report, continuing to describe fatigue. Her blood counts were normal at that time, and she had no history of prior blood transfusions. She then received multiple intravenous infusions containing hydrogen peroxide, ozone, and colloidal silver, the latter also in the form of Argentyn 23. Her total administered dose of parenteral colloidal silver was 623 mg. Three months later, she was found to have a hemoglobin concentration of 7.1 g/dL, with a total leukocyte count of 2.6 × 10^9^/L and 40% neutrophils. The platelet count was 28.8 × 10^9^/L. The uncorrected reticulocyte count was 1.1% (absolute reticulocyte count 0.663 cells/µL) with normal serum LDH and haptoglobin values. Her serum ferritin value was greatly elevated a 2063 ng/L, and the serum silver concentration was 220 mcg/L. Her initial serum copper level was < 5 mcg/L, and after several weeks of receiving oral copper supplements, rose only to 11 mcg/L. Her serum zinc concentration was not initially measured and following oral copper therapy was later found to be 127 mcg/dL (normal value 60‐130 mcg/dL). She received a total of eight units of blood over the next several weeks to maintain a satisfactory hemoglobin concentration. She also received a course of treatment with Succimer, without any effect on reducing her elevated serum silver level. She declined apheresis. Her leukopenia gradually resolved, with the final blood count available to us indicating a hemoglobin concentration of 10.6 g/dL, total leukocyte count of 5.1 × 10^9^/L with 68% neutrophils, and a platelet count of 224 × 10^9^/L. At that time, the markedly elevated serum ferritin value had fallen slightly to 1827 ng/L. She was advised to continue oral copper therapy but she declined further follow‐up.

### 2.3 Case 3

A 46‐year‐old woman sought medical attention at the same Wellness Center described for Case 1 and Case 2 and also with symptoms of chronic fatigue. Her physical examination was normal, and she had no past history of anemia or liver disease. Initial laboratory examination revealed a hemoglobin concentration of 13.3 g/dL (MCV 96 fL) with a total leukocyte count of 4.3 × 10^9^/L and platelet count of 281 × 10^9^/L. A differential white blood cell count was unremarkable, with 51% neutrophils. Additional chemistry values showed normal liver and renal function values. Serologic panels for multiple hepatic viral diseases gave normal results and serologic tests for Lyme disease and a Western blot study were also normal, as in Case 1.

Nevertheless, she was also informed that she had Lyme disease and received multiple intravenous infusions of hydrogen peroxide, ozone, and colloidal silver, in a similar fashion to our Case 1 and Case 2, but we were unable to obtain records of her total administered dose of colloidal silver. Three months later, her hemoglobin concentration had fallen to 7.8 gm/dL (MCV 109 fL) with a total leukocyte count of 3.4 × 10^9^/L and 36% neutrophils. The platelet count was 420 × 10^9^/L). The uncorrected reticulocyte count was 0.8% (absolute reticulocyte count 0.416 cells/µL) and serum erythropoietin value only mildly increased relative to the degree of anemia, at 68.7 IU (normal value, at a normal hemoglobin concentration, 3.7‐36 IU). She received multiple blood transfusions, but remained anemic and clinically became progressively very ill and was ultimately admitted to the hospital 2 months later, and there referred for hematology consultation. Her admission laboratory evaluation showed an elevated alkaline phosphatase value of 792 IU/L (normal range 44‐147 IU/L), and ALT and AST values of 416 U/L and 487 U/L, respectively (normal range 8‐40 U/L), and a total bilirubin concentration of 2.5 mg/dL. These transaminase levels continued to rise and peak at 1,200 and 670 U/L, respectively, with a total bilirubin of 4.5 mg/dL. All of these elevated laboratory values then gradually declined. Her serum silver level was 210 mcg/dL and initial copper level below accurate laboratory quantitation and reported as <5 mcg/dL Serum haptoglobin concentration was 109 mg/dL (normal range, 30‐300 mg/dL). Imaging studies indicated a slightly enlarged spleen at 13 cm in length. She required additional blood transfusions. Her admitting serum ferritin value, which was normal precolloidal silver infusion, was now 332 ng/dL, and it continued to rise and peak at 1278 ng/dL (normal range for women, 12‐150 ng/dL).

She received oral copper supplementation daily, as Case 1 and Case 2 did, once silver poisoning was documented, but her serum copper level rose only to 19 mcg/L after weeks of administration. She refused to consider apheresis. A liver biopsy revealed mild, patchy portal, and lobular inflammation with scattered apoptotic hepatocytes and thought consistent with drug toxicity effect. Her abnormal liver function ultimately abated over several weeks without any specific therapy, and her elevated platelet count returned to normal. Nevertheless, she remained with significant anemia at discharge, but declined continuing follow‐up by the hematologist.

## 3 DISCUSSION

The hematological effects of intravenous colloidal silver infusions were studied by the Nobel Laureate, Dr George H. Whipple. In 1930‐1931, he infused multiple dogs with colloidal silver in a fashion similar to our three patients.^9^ Prior animal studies suggested to him that the causal mechanism for silver‐induced anemia might be induction of bone marrow aplasia, a model he was seeking in the study of human aplastic anemia. His dogs all ultimately died with marked anemia but with a hypercellular bone marrow appearance on repeat examinations. He thought the mechanism of the anemia had some association with hemolysis by the gross serum and urine appearance but he did not describe the peripheral blood film morphology. It is possible the dogs had manifest liver pathology, as our Case 3, causing the visual changes he observed in the plasma and urine, and not significant hemolysis.

In more current literature, that copper and zinc deficiency may both occur with silver poisoning has been commented upon.^10^ It is known that zinc and copper compete with each other for access to a common relatively weak excretion pathway and that excess oral zinc administration may cause copper deficiency anemia.^11^, ^12^, ^13^, ^14^, ^15^ It appears from our three patients, that infused colloidal silver has no significant excretion pathway, as the markedly elevated serum silver levels remained constant over many weeks. However, its presence likely facilitates persistent excess urinary losses of both copper and zinc, and particularly for copper, into both the bile and urine. Copper deficiency is known to produce significant anemia and leukopenia, even simulating myelodysplastic syndromes (MDS) by the abnormal peripheral blood and bone marrow cellular morphology; increased hematogones, which may be confused with blast forms, were noted in the bone marrow of our Case 1.^15^

The anemia of colloidal silver poisoning is presumably promoted by the resulting profound copper deficiency and attributed to ineffective myelopoiesis with a cellular marrow, as present in MDS and in our Case 1.^13^, ^14^, ^15^, ^16^ Ringed sideroblasts have also been noted in the bone marrow with copper deficiency‐induced anemia but were not present in our patient. She did manifest a small number of spherocytes in the peripheral blood but hemolysis did not appear to significantly contribute to the anemia. It may simply have been a consequence of the more elevated serum silver level relative to those measured in our other two cases.

Severe copper deficiency is no longer an unusual diagnosis in the modern era, now occasionally occurring shortly following bariatric Roux‐en‐Y surgery with anemia and leukopenia as a consequence. Short‐term parenteral administration of zinc and copper salts have been used in this setting, as effective therapy.^17^ In our Case 1, it appeared that the massive blood silver level effectively accelerated excretion of virtually all of the initially administered oral copper. Providing excess oral copper, in this circumstance, may have retarded excessive loss of zinc, which competes with copper for excretion, and eliminated the need for supplemental zinc. We did not perform a zinc level prior to initiating oral copper therapy in this patient but later, prior to apheresis, it was 96 mcg/dL (normal value, 60‐120 mcg/dL). The serum zinc may initially have been low, but could have become normal consequent to the prolonged administration of excess oral copper. This would be unlike the bariatric surgery mediated mineral losses.

## 4 CONCLUSION

As this report illustrates, the use of infused colloidal silver as a primary or adjunctive therapy to treat alleged Lyme disease or other systemic infections must be condemned as both ineffective and with predictable long‐term toxicity. Silver administered by the intravenous route is more toxic than that associated with oral preparations because of both higher dose and the apparent inability of the body to excrete this material, resulting in continuous prolonged and profound intravascular colloidal silver exposure. Copper and possibly zinc losses are accelerated by the presence of circulating colloidal silver, and reduction of particularly serum copper may cause severe anemia as a consequence. The resulting anemia creates an unusual peripheral blood erythrocyte morphologic pattern similar to that of isolated copper deficiency and simulating MDS. However, it may have a slight direct hemolytic component evident at the higher colloidal silver levels present in Case 1, presumably explaining the very small number of spherocytes, without apparent clinical significance. Serum ferritin values rapidly increase following colloidal silver poisoning and may represent an inflammatory component or acute phase stimulus.

The only effective therapy to significantly reduce the circulating colloidal silver load, is with apheresis. We found only a single patient previously so treated and reported in the medical literature.^6^ In that case, the greatly elevated serum silver level dropped substantially after a single exchange. However, the patient died with neurological complications of the silver toxicity before additional apheresis sessions could be accomplished.

Serial apheresis is clearly useful therapy, as demonstrated with the outcome of Case 1, in contrast with the need for repeated transfusions and persistent anemia despite oral copper supplementation present in Case 2 and Case 3. This, even though their serum silver levels were significantly less than those measured in Case 1. Apheresis should be supplemented with constant oral copper administration in order to maintain a satisfactory hemoglobin concentration.

Modern apheresis with albumin rather than plasma substitution is a very safe procedure but the intravascular silver reduction increment lessens with each successive exchange. Nevertheless, it appears that the major reduction in circulating silver accomplished by apheresis eliminates need for supportive blood transfusions and may reduce the later appearance of argyrosis. The required dose of supplemental oral copper is also clearly reduced. However, the ultimate potential consequences of persistently elevated intravascular colloidal silver levels, such as argyria, or renal dysfunction, are uncertain and will require longer‐term observation of the patient group described here.

We reported full documentation for all three cases to the Texas Medical Board for review, and ultimately, the initial treating physician agreed to voluntarily surrender his Texas medical license in lieu of further disciplinary proceedings.

## CONFLICT OF INTEREST

Each of the authors declare that there is no conflict of interest regarding the publication of this paper.

## AUTHOR CONTRIBUTIONS

EAN: involved in hematology consulting and treating physician for the patient described as Case 1 and prepared the manuscript and photographs.KRB: involved in hematology consulting and treating physician for the patients described as Cases 2 and 3 and reviewed the manuscript.and DWP: helped, as a toxicologist, with literature research concerning chelation therapy and silver toxicity and calculations as to the silver content of the colloidal silver product, Argenten 23, discussed in the manuscript. He also reviewed the manuscript.
